# 84084 Team Science to maximize rapid collection and analyses of biosamples from patients with Covid-19

**DOI:** 10.1017/cts.2021.654

**Published:** 2021-03-30

**Authors:** Sharon M Moe, Brooke Patz, Yunlong Liu, Christie Orschell, Andy Yu, Scott Denne, Peter Embi, Tatiana Foroud

**Affiliations:** Indiana University, Embi also from Regenstrief Institute

## Abstract

ABSTRACT IMPACT: Indiana CTSI Team Science to maximize rapid collection, analyses and dissemination of biosamples collected from patients with Covid-19 to provide preliminary data for grant applications on the pathogenesis and outcomes of patients with Covid-19. OBJECTIVES/GOALS: When Covid-19 hit Indiana in April, there was an immediate need to respond rapidly to coordinate research across our healthcare systems. The CTSI became a point of contact for coordinating research endeavors including activation of clinical trials and use of precious samples from patients with Covid-19 to maximize preliminary data for grants. METHODS/STUDY POPULATION: The Indiana CTSI coordinated collection of biospecimens at multiple hospitals using in person and remote consenting via telephone or on a smartphone utilizing a QR code. We also retrieved existing samples from the Indiana Biobank previously collected for future research and from subject positive for Covid-19 by search of the linked electronic health record (EHR). A total of 224 subject samples (7 children, 36 previously collected, and 6 with both acute and recovered specimens) were obtained over a four month period. Our CTSI cores ran varied analyses collated to a single database, linked to the EMR for use as preliminary data for grant applications to avoid redundancy of measures on limited samples. RESULTS/ANTICIPATED RESULTS: The 224 subject samples were used for whole exome DNA sequencing, RNA seq, analyses of 48 plasma cytokine/chemokines by multiplex analyses, and PBMC isolated for culture and assessment of secreted cytokines. The clinical data were linked and included demographics, hospitalization length of stay and need for mechanical ventilation, max and min oxygen levels, liver function tests, IL-6, D-dimer, CRP, LDH, and ferritin, need for dialysis, and echocardiography. Additional clinical data were available upon request. A survey was sent to our CTSI email to query for potential interest in the data with 87 inquiries, and to date 46 investigators have requested data and/or additional samples. DISCUSSION/SIGNIFICANCE OF FINDINGS: During the first surge of Covid-19, the CTSI coordinated analyses for the dissemination of results for use by CTSI investigators to minimize duplication of assays and increase availability. The collaboration of research coordinators, biobank, research cores, and informatics demonstrates the power and agility of team science in the Indiana CTSI.

